# Quantitative Analysis of Glassy State Relaxation and Ostwald Ripening during Annealing Using Freeze-Drying Microscopy

**DOI:** 10.3390/pharmaceutics14061176

**Published:** 2022-05-30

**Authors:** Tigran Kharatyan, Srikanth R. Gopireddy, Toru Ogawa, Tatsuhiro Kodama, Norihiro Nishimoto, Sayaka Osada, Regina Scherließ, Nora A. Urbanetz

**Affiliations:** 1 Department of Pharmaceutics and Biopharmaceutics, Kiel University, 24118 Kiel, Germany; tkharatyan@pharmazie.uni-kiel.de (T.K.); rscherliess@pharmazie.uni-kiel.de (R.S.); 2 Pharmaceutical Development, Daiichi-Sankyo Europe GmbH, 85276 Pfaffenhofen an der Ilm, Germany; nora.urbanetz@daiichi-sankyo.eu; 3 Formulation Technology Research Laboratories, Daiichi Sankyo Co., Ltd., Hiratsuka 254-0014, Japan; toru.ogawa@daiichi-sankyo.eu (T.O.); kodama.tatsuhiro.s8@daiichisankyo.co.jp (T.K.); nishimoto.norihiro.fy@daiichisankyo.co.jp (N.N.); osada.sayaka.av@daiichisankyo.co.jp (S.O.)

**Keywords:** lyophilisation, annealing, devitrification, supercooling

## Abstract

Supercooling during the freezing of pharmaceutical solutions often leads to suboptimal freeze-drying results, such as long primary drying times or a collapse in the cake structure. Thermal treatment of the frozen solution, known as annealing, can improve those issues by influencing properties such as the pore size and collapse temperature of the lyophilisate. In this study we aimed to show that annealing causes a rearrangement of water molecules between ice crystals, as well as between the freeze-concentrated amorphous matrix and the crystalline ice phase in a frozen binary aqueous solution. Ice crystal sizes, as well as volume fractions of the crystalline and amorphous phases of 10% (*w*/*w*) sucrose and trehalose solutions, were quantified after annealing using freeze-drying microscopy and image labelling. Depending on the annealing time and temperature, the amorphous phase was shown to decrease its volume due to the crystallisation of vitreous water (i.e., glassy state relaxation) while the crystalline phase was undergoing coarsening (i.e., Ostwald ripening). These results allow, for the first time, a quantitative comparison of the two phenomena. It was demonstrated that glassy state relaxation and Ostwald ripening, although occurring simultaneously, are distinct processes that follow different kinetics.

## 1. Introduction

Freeze-drying is a commonly used method to stabilise pharmaceutical products [1]. The choice of excipients plays a decisive role in the outcome, since material properties such as the glass transition temperature ($T_{g}$) and collapse temperature ($T_{c}$), have an impact on the drying process [2,3]. Temperatures above $T_{c}$ during primary drying lead to softening of the lyophilisate to the point of breakdown of the structure, i.e., collapse [4,5]. However, low drying temperatures can greatly increase the duration of a freeze-drying cycle [6]. Therefore, an understanding of such phenomena is essential for process optimisation in the field of lyophilisation.

**Figure 1:**
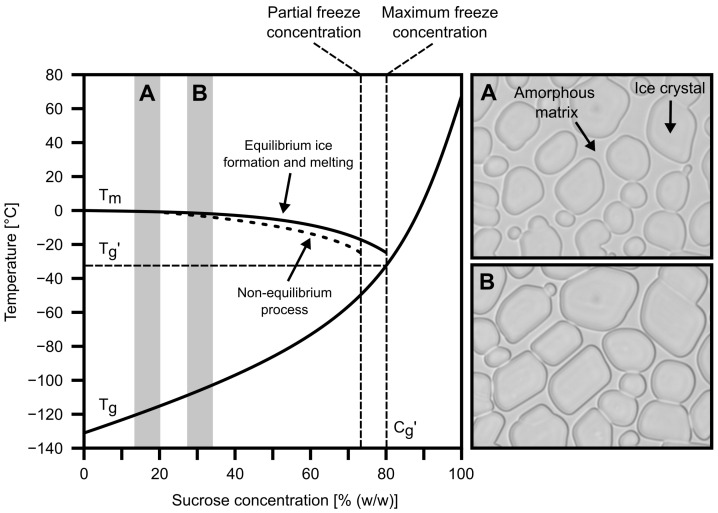
Simplified phase diagram of a sucrose-water system (**left**) and exemplary microscopy pictures (**right**) of a cooled sucrose solution (based on Zhao & Takhar [10]).

Frequently used excipients for amorphous protein drug products include glass-formers such as sucrose and trehalose [7]. Freezing of an aqueous solution with a glass former leads to freeze-concentration, a microscopic separation of the mixture into its individual components. When the solution is cooled below its liquidus temperature, water molecules begin to precipitate and form pure ice crystals. The second component concentrates and forms a continuous amorphous phase, together with the non-precipitated residual water [8]. The lower the temperature, the more the phases separate, i.e., the more crystalline ice phase is formed, while the solute continues to accumulate in the amorphous phase. This effect is based on the temperature-dependent chemical potential of different molecule species forming hydrogen bonds with each other. Ice formation occurs as long as the chemical potential of water molecules with hydrogen bonds in ice is lower than that of water molecules with hydrogen bonds with solute molecules [9]. Under equilibrium conditions, this process follows the liquidus line or melting temperature $T_{m}$. The phase separation ends when the solute in the amorphous matrix reaches its maximum freeze-concentrated concentration ($C_{g}$${}^{\prime }$) and contains the minimum residual water content ($W_{g}$${}^{\prime }$) that can be achieved via freeze-concentration [8]. Such behaviour is illustrated in Figure 1 with the material-specific phase diagram of water and sucrose, which shows the transition temperatures of the binary system as a function of its mass fractions.

According to the phase diagram of the sucrose-water system, $T_{g}$ depends on the ratio of water to the solute. To be more precise, transitional events such as glass transition are linked to the residual water content of the freeze-concentrated matrix. It follows that a frozen glass-forming solution in its maximum freeze-concentrated state should exhibit the highest $T_{g}$, denoted as $T_{g}$${}^{\prime }$. However, rapid freezing due to supercooling of an aqueous solution can facilitate a non-equilibrium process and lead to partial freeze-concentration, in which a residual amount of water does not crystallise, but remains in the matrix in non-crystalline form as vitreous water [11,12]. In this case, an increase of vitreous water in the amorphous matrix decreases its solute concentration and therefore reduces $T_{g}$${}^{\prime }$.

Measurements of glass-forming carbohydrates by means of differential scanning calorimetry (DSC) show that $T_{g}$${}^{\prime }$ is not only material-dependent, but also affected by the thermal history of a sample [11,12]. If equilibrium freezing cannot be guaranteed, post-freezing annealing can be used to allow the amorphous matrix to reach its equilibrium state and complete freeze-concentration [8].

Annealing in freeze-drying describes an optional process step between freezing and primary drying. In this procedure, the temperature of the frozen sample is raised again, without thawing it completely. The temperature ramp, annealing temperature ($T_{a}$) and annealing duration can be selected. Afterwards, the material is cooled once again and the lyophilisation process continues. This technique is often used for process optimisation, since it is commonly observed that, for example, primary drying times can be shortened [5,13]. In addition, studies show that annealing can help to maintain the stability of proteins in pharmaceutical formulations during freeze-drying [14,15]. Two phenomena may be observed during the annealing of a binary aqueous solution: Ostwald ripening and glassy state relaxation.

Ostwald ripening or migratory recrystallisation describes the redistribution of mass between crystals and is based on the Gibbs–Thomson effect [16]. Small particles have stronger surface curvature and thus exhibit larger surface free energy than bigger particles. As a result, their solubility is increased to such an extent that a diffusive mass flow from small to large particles takes place. Consequently, small particles shrink in favour of larger particles until they reach a critical nucleus size and completely dissolve [17,18].

Glassy state relaxation refers to the devitrification of non-crystalline water within the amorphous solute-water matrix. Annealing can convert this water into the crystalline phase, thereby decreasing the residual water content of the amorphous matrix and increasing its $T_{g}$${}^{\prime }$ [19,20]. Experiments using X-ray micro-computed tomography (µ-CT) show that glassy state relaxation progresses with increasing annealing time, and that its rate is dependent on $T_{g}$${}^{\prime }$ of the solution [21].

The motivation for this work was to provide comprehensive insights about ice formation in pharmaceutical solutions and to provide the basis for understanding how annealing, depending on temperature and time, influences the properties of the frozen solution and affects the subsequent steps in the lyophilisation cycle. Other publications have also dealt with these issues, but have only been able to analyse Ostwald ripening and glassy state relaxation individually, or at least with separate methods. In contrast, in this study we utilise freeze-drying microscopy to observe both phenomena in the same sample and present a method that allows for easy evaluation while remaining accurate and being much more cost-effective compared to techniques such as µ-CT. Glassy state relaxation was evaluated by determining volume fractions under different annealing conditions. Theoretical calculations were performed to confirm whether the experimentally determined values were plausible. Finally, the average ice crystal sizes during annealing were measured to allow a comparison between Ostwald ripening and glassy state relaxation.

## 2. Materials and Methods

### 2.1. Materials

A 10% (*w*/*w*) sucrose solution with NF-grade d-(+)-sucrose from Merck Millipore (Burlington, MA, USA) and a 10% (*w*/*w*) trehalose solution with NF-grade d-(+)-trehalose from VWR International (Radnor, PA, USA) were prepared with purified water. Samples were filtrated with 0.22 µm Millex^®^-GV syringe filters from Merck Millipore and stored at 5 °C until use.

### 2.2. Freeze-Drying Microscopy

Freeze-drying microscopy (FDM) is traditionally used to determine $T_{c}$. FDM uses a freeze-drying stage system to set temperature and pressure, similar to the process conditions in a lyophiliser. The sample is placed between two glass plates, which are kept at a distance by means of a spacer. Inside the freeze-drying stage system, the glass plates lie on top of a silver block, which can be electrically heated or cooled with liquid nitrogen. The freeze-drying stage system is barometricallyisolated so that a vacuum can be generated with a vacuum pump. During drying, the temperature is increased incrementally until structural changes of the sample, i.e., collapse, can be observed at the sublimation front.

**Figure 2:**
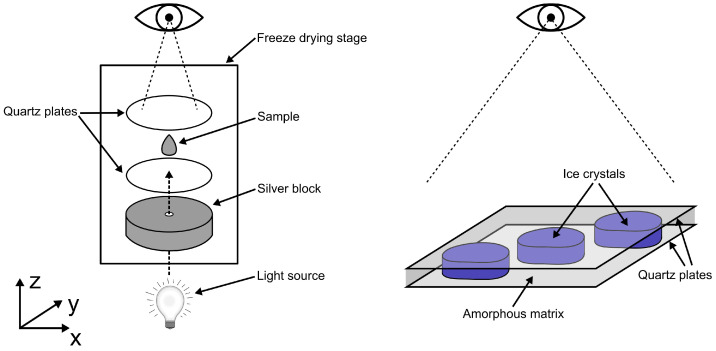
Schematic illustrations of the freeze-drying stage (**left**) and microscopic cutout of ice crystals (blue) embedded in the amorphous matrix (grey) in the experimental setup (**right**).

For each experiment, 0.3 µL of 10% (*w*/*w*) sucrose or trehalose solution was pipetted onto a quartz plate and covered with an identical plate (d = 16 mm; calculated sample height ≈ 15 µm). The spacer was omitted in order to detect and quantify individual structures more clearly, since the layering of crystals is inhibited by the restriction of the sample height. The quartz plates were placed on the silver block of the freeze-drying stage (FDCS196; Linkam Scientific Instruments Ltd., Redhill, UK) and the lid was screwed shut. The samples were first cooled to −50 °C at 50 °C/min in order to provoke non-equilibrium freezing, then heated to the respective annealing temperature (−4 °C and −6 °C) at 10 °C/min and held for the annealing time (30 min, 60 min, 120 min, 240 min, 360 min and 480 min) at ambient pressure. During this period, images were taken every 10 min with the 20× lens of the light microscopy system (microscope: Axioscope 5; camera: Axiocam 305 color; Carl Zeiss, Germany). Finally, the samples were again cooled to −50 °C at 10 °C/min and observed through the 50× lense. Different magnifications were used (1) to accommodate the richness of detail for fraction determination and (2) to provide more data on the morphological properties of ice crystals. Using the sample holder, the sample was moved in the x- and y-planes so that images of different areas could be obtained. This is especially important if the solute is unevenly distributed due to the effects of convective cryo-concentration. Figure 2 shows the schematic setup of the FDM experiments.

### 2.3. Picture Labelling and Evaluation

The proposed method presumes that objects adopt cylindrical shapes, i.e., the crystalline phase has a similar contact area with both quartz plates (x- and y-plane) and no curvature along the z-axis (see Figure 2). This assumption is only valid if the average crystal size is sufficiently large due to the progression of Ostwald ripening. Otherwise, small ice crystals can form spherical structures and lead to ambiguous results. Under this prerequisite, the volume fraction $\phi_{i}$ (Equation (1)) is equal to the area fraction, since the volume $V_{i}$ is obtained from the contact area $A_{i}$ of the crystalline phase to the quartz plate and the height *h* between the quartz plates.

(1) $$\phi_{i}=\frac{V_{i}}{\sum_{j}V_{j}}=\frac{A_{i}\cdot h}{\sum_{j}A_{j}\cdot h}$$

Processing of FDM data is necessary because light microscopy images are prone to artefacts. For this purpose, the phases in the images were filled with different colours (crystalline ice = white; amorphous matrix = black). The observable interface between ice crystals and the amorphous phase is formed due to the slight curvature of the ice crystals along the z-axis and the differing optical densities of the phases. The light from the light source is refracted at different angles at these points and is visible in the microscope as a dark border around the ice crystals. In this experimental setup, however, the area of the interface turns out to be so narrow that cylindrical ice crystals are assumed for analysis.

**Figure 3:**
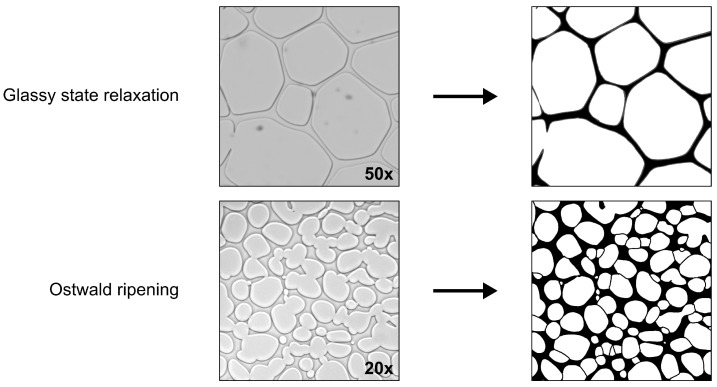
Labelling and evaluation of FDM pictures for the determination of glassy state relaxation and Ostwald ripening. Images for glassy state relaxation were taken after refreezing an annealed sample to −50 °C, whereas Ostwald ripening data were gathered at the annealing temperature. Ice and matrix fractions are shown in white and black, respectively.

To account for a possible unequal distribution of the solute, 3 images were evaluated for each condition. Figure 3 shows a cutout example for the labelling and evaluation process.

Volume fractions were identified from labelled images with 50× magnification in order to keep as much detail as possible. After labelling, ImageJ (NIH, Bethesda, MD, USA) was used to assign pixels to a fraction according to their colour (crystalline ice = white; amorphous matrix = black). A change in the amount of pixels associated with a phase would therefore be an indication of a shift in the fractions of the binary system. In order to determine morphological data (e.g., average ice crystal size), labelled images during the annealing process (30 min, 60 min, 120 min, 240 min, 360 min and 480 min) from 20× magnification were evaluated with the particle analysis function in ImageJ.

## 3. Results and Discussion

FDM experiments were successfully performed and the generated images were labelled. In general, the higher the annealing temperature and/or the longer the annealing time, the easier the evaluation with this method, since small ice crystals are comparatively more time-consuming to label than larger ones. In addition, higher annealing temperatures allow a more uniform distribution of the solute, which may show local concentration gradients due to cryo-concentration.

### 3.1. Effect of Annealing on Volume Fractions

**Figure 4:**
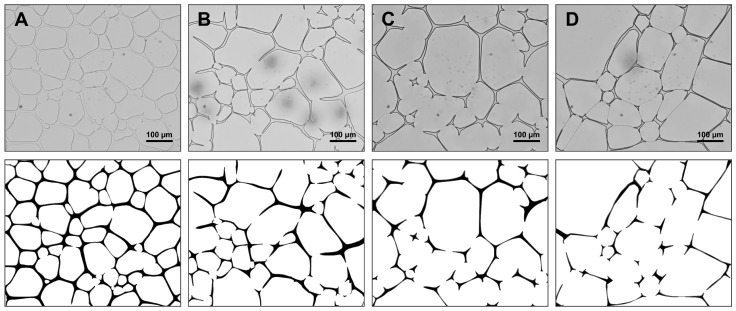
FDM pictures with 50× magnification of a 10% (*w*/*w*) sucrose solution after freezing to −50 °C, subsequent annealing at −6 °C for different times ((**A**) = 60 min, (**B**) = 120 min, (**C**) = 240 min, (**D**) = 360 min) and recooling to −50 °C (**upper row**). Corresponding labelled pictures show crystalline phase in white and amorphous matrix in black (**lower row**).

In order to evaluate glassy state relaxation, volume fractions were determined for freeze-concentrated sucrose and trehalose solutions after various annealing conditions. A change in volume fractions is associated with relocation of water from the amorphous to the ice crystal phase due to devitrification. Figure 4 shows exemplary microscopic images after samples were annealed at −6 °C for different times (A = 60 min, B = 120 min, C = 240 min, D = 360 min) and refrozen to −50 °C with corresponding labelled versions. Changes between different annealing times can already be seen with the naked eye. Longer annealing durations resulted in coarsening of the crystalline ice phase (white area) as the number of ice crystals decreased and the average size of ice crystals increased. At the same time, the amorphous phase (black area) decreased in volume with increasing annealing time.

**Figure 5:**
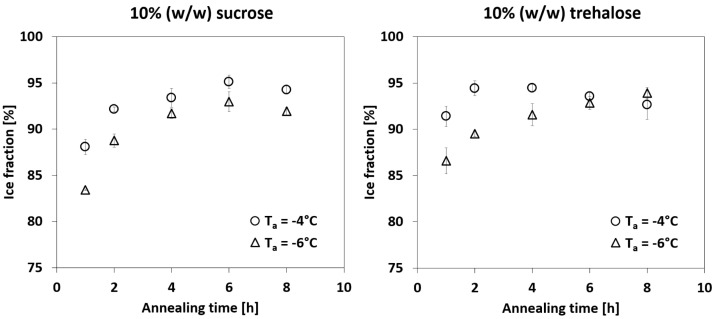
Ice crystal fraction as a function of annealing time for different annealing temperatures of 10% (*w*/*w*) sucrose and trehalose solutions. Standard deviations were calculated from triplicates.

In principle, it was observed that the longer the annealing time, the larger the ice fraction. This process progressed until a plateau was reached, indicating that the volume reduction of the amorphous phase was completed. An increase in annealing temperature meant that the plateau was reached faster. Figure 5 shows the quantified ice fractions for different annealing conditions.

After rapid freezing, vitreous water was present in the amorphous phase, which increased the volume of the latter. During annealing, this water was gradually devitrified and accumulated in the crystalline ice phase. As a result, the volume of the amorphous phase decreased and the solute in the matrix was concentrated further. Different annealing durations were necessary depending on the annealing temperature to reach the state of maximum freeze-concentration. This is indicated by the plateau of the ice fraction towards longer annealing times. The relaxation rate of the amorphous phase has been reported to depend on the difference between the annealing temperature and $T_{g}$${}^{\prime }$ of the solute (*T* − $T_{g}$${}^{\prime }$) [22], which is consistent with the results of this study. In the experimental setup with 10% (*w*/*w*) sucrose, this process appeared to be completed after 4 h to 6 h at the latest for annealing temperatures of −4 °C and −6 °C. A similar behaviour was also observed for the 10% (*w*/*w*) trehalose solution. However, less vitreous water seemed to be present as a starting condition. The plateau was reached already after annealing for 2 h at −4 °C. Nakagawa et al. [21] determined with µ-CT analysis that a 20% (*w*/*w*) sucrose solution required almost 20 h of annealing at −5 °C to complete glassy state relaxation. The shortest annealing time used in the study by Nakagawa et al. was 6 h. The present study focuses on annealing times and excipient concentrations that are more frequently used in lyophilisation cycles. Additionally, an identical sample preparation and quantification technique were used to observe the volume change in the amorphous phase and the coarsening of the crystalline ice phase. In contrast, µ-CT analysis of small structures such as ice crystals is problematic due to resolution issues, therefore requiring additional tools such as optical microscopes to determine geometrical properties such as ice crystal sizes.

### 3.2. Theoretical Volume Fractions of a Freeze-Concentrated Solution

To validate the FDM method, volume fractions of the maximum freeze-concentrated solution were compared with theoretical calculations according to values from literature. In terms of $C_{g}^{\prime }$ and $W_{g}^{\prime }$, trehalose and sucrose exhibit similar properties [23]; therefore, the calculations were performed for sucrose only. Table 1 shows the derived volume fractions.

For sucrose solutions (20–70%), Ross and Karel [24] found that about 20% (*w*/*w*) residual water remained in the maximum freeze-concentrated amorphous phase. For an initial 10% (*w*/*w*) solution, this constitutes a mass fraction of 0.125 for the amorphous phase. Although the density of ice at a temperature of −50 °C is known [25], such data are not available for an 80% (*w*/*w*) sucrose mixture. Therefore, the density at 20 °C was used for the calculation. Potential material contraction/expansion and consequent density changes due to low temperatures were disregarded. Volume fractions of 0.915 for the ice phase and 0.085 for the amorphous phase were obtained, i.e., 91.5% of the total volume was occupied by ice in the maximum freeze-concentrated state of the 10% (*w*/*w*) sucrose solution and the remaining 8.5% by the amorphous matrix.

The comparison was made with the averaged volume fractions obtained from experiments. For this purpose, data from the longest experiments (8 h) were taken as a basis, since they allowed for most glassy state relaxation to occur. This gave an experimental volume fraction of 93.03% ± 1.11% for the ice phase and 6.96% ± 1.58% for the amorphous phase. These results are in good agreement with the theoretical values. Slight deviations from the calculations can be explained by the fact that (1) the density of an 80% (*w*/*w*) sucrose solution at −50 °C is not known, (2) the ice crystals in the experiments exhibited curvature along the z-axis, and (3) convective phenomena were regarded only partially.

### 3.3. Relation between Devitrification and Recrystallisation

**Figure 6:**
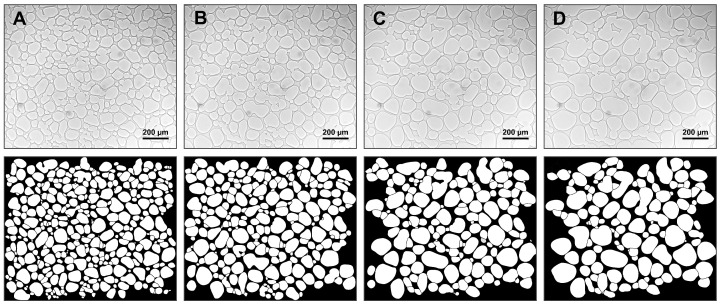
FDM pictures with 20× magnification of a 10% (*w*/*w*) sucrose solution during annealing for different times ((**A**) = 60 min, (**B**) = 120 min, (**C**) = 240 min, (**D**) = 360 min) at −6 °C (**upper row**). Corresponding labelled pictures show crystalline phase in white and amorphous matrix in black (**lower row**).

The evaluation of Ostwald ripening is based on the change in the average ice crystal size over time. Figure 6 shows exemplary microscopic images during anealing (A = 60 min, B = 120 min, C = 240 min, D = 360 min) with corresponding labelled versions. Ice crystals that were not completely in the field of view of the microscope were excluded from the evaluation; otherwise the size distribution would have been distorted. This effect was especially prominent when a lot of coarsening had occurred. Progressive coarsening of the crystalline phase over time was observed. The number of particles decreased, whereas the average size increased over time.

**Figure 7:**
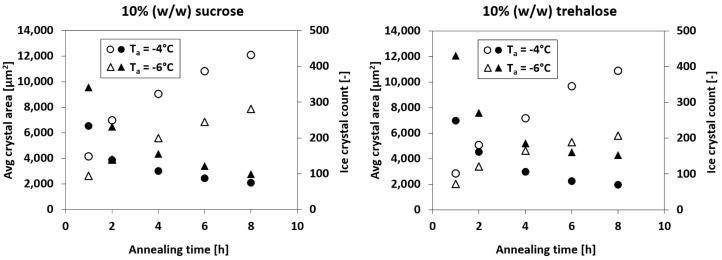
Average ice crystal size (open symbols) and ice crystal count (filled symbols) as a function of annealing time for different annealing temperatures of 10% (*w*/*w*) sucrose and trehalose solutions.

Figure 7 shows the average ice crystal size for different annealing temperatures as a function of annealing time. It should be noted that the data were recorded during annealing. This means that an annealing temperature of −4 °C will lead to more water in the amorphous phase than at −6 °C due to the temperature dependency of ice formation. Therefore, higher annealing temperatures will underestimate the final ice crystal size during recooling more than lower annealing temperatures. The trend, however, remains the same.

A steady and temperature-dependent increase in the average ice crystal size was observed, with trehalose coarsening slower than sucrose. The differences in the coarsening rates can be explained with the water mobility in each solution. Klinmalai et al. [27] assumed that water molecules interact in two different ways with saccharides – low interaction for so-called free water and stronger interaction for so-called associating water. The ratio of free water to associating water will thus have an impact on the net water mobility of the solution. In the work of Hagiwara et al. [18], ^1^H spin-spin relaxation times, which show correlations to water mobility [28], indicated similar results. Additionally, experimental data on Ostwald ripening for various mono- and disaccharides were collected. It was found that a linear correlation exists between the Sauter mean diameter and the annealing time. This correlation was demonstrated for various annealing temperatures up to an annealing duration of more than 12 h.

No plateau was observed in this study, even after annealing for 8 h at −4 °C. Over the entire observation period, a linear increase in the average crystal area was observed for −4 °C and −6 °C. Comparing these results with the data for glassy state relaxation (see Figure 5), it is evident that both processes are distinct. This is in agreement with the finding of Nakagawa et al. [21]. However, the advantage of the present work consists in the application of the same technique to observe both phenomena, namely, glassy state relaxation, as well as Ostwald ripening.

The importance of both phenomena for the optimisation of the lyophilisation process can be explained as follows. During primary drying, water molecules from precipitated ice crystals sublimate and are removed from the lyophilisate. The space left behind forms the pores within the lyophilisate, which in turn influence the sublimation resistance of water vapor [5]. Annealing increases the average ice crystal size, therefore reducing the sublimation resistance and increasing the sublimation rate. Influencing ice crystal sizes can therefore potentially shorten a lyophilisation cycle. The composition of the amorphous phase during drying defines its $T_{g}$${}^{\prime }$. Since $T_{c}$ is generally 2 °C to 5 °C higher than $T_{g}$${}^{\prime }$ [29], control over the composition of the amorphous phase during lyophilisation is essential in order to inhibit undesired effects such as collapse.

## 4. Conclusions

Freeze-drying microscopy was used to observe glassy state relaxation and Ostwald ripening of sucrose and trehalose solutions during annealing. FDM images were taken from different areas of the sample, labelled according to the crystalline and amorphous fractions and evaluated. It was shown that both glassy state relaxation and Ostwald ripening advanced with annealing time and temperature. However, a plateau could be only observed for glassy state relaxation over time. In the case of −4 °C, a plateau was reached after about 4 h, whereas an annealing temperature up to −6 °C required between 4 h to 6 h. No plateau was detected for Ostwald ripening over any of the annealing temperatures and times. Instead, an increase in the average ice crystal size was observed for all annealing conditions. Glassy state relaxation and Ostwald ripening jointly influence the evolution of the ice crystal morphology in a frozen binary aqueous solution. The former affects the volume fractions, whereas the latter determines the average crystal sizes. Initially they occur simultaneously, but at some point the devitrification of vitreous water in the amorphous phase is completed, although coarsening continues. These differences in behaviour are expected, since Ostwald ripening depends on the local curvature of the particles, whereas glassy state relaxation is driven by the enthalpy of the amorphous phase. Nevertheless, the findings of this work allow us to assess the observation times of both phenomena. As a next step, research can be targeted towards finding a correlation between macroscopic observations of lyophilisates, such as shrinkage, and the two phenomena discussed in this work. The results of such experiments could then shed light on which mechanism is responsible for changes in the product during freeze-drying. A fundamental understanding and quantification of Ostwald ripening and glassy state relaxation would thus lead to the determination of annealing conditions that could be used specifically to optimise the freeze-drying process.

## Figures and Tables

**Table 1:** Derived volume fractions of a maximum freeze-concentrated 10% (*w*/*w*) sucrose solution, based on published data.

Parameter	Ice Phase	Amorphous Phase	Source
Mass fraction (-)	0.875	0.125	[24]
Density (kg/m^3^)	0.921 (at −50 °C)	1.414 (at 20 °C)	[25,26]
Volume fraction (-)	0.915	0.085	-

## Data Availability

Data available on request.
